# Myocardial Impact of NHE1 Regulation by Sildenafil

**DOI:** 10.3389/fcvm.2021.617519

**Published:** 2021-02-22

**Authors:** Daiana S. Escudero, Néstor G. Pérez, Romina G. Díaz

**Affiliations:** Centro de Investigaciones Cardiovasculares “Dr. Horacio E. Cingolani”, Facultad de Ciencias Médicas, Universidad Nacional de La Plata, La Plata, Argentina

**Keywords:** NHE1, sildenafil, intracellular pathways, cardiac mechanism, PDE5A

## Abstract

The cardiac Na^+^/H^+^ exchanger (NHE1) is a membrane glycoprotein fundamental for proper cell functioning due its multiple housekeeping tasks, including regulation of intracellular pH, Na^+^ concentration, and cell volume. In the heart, hyperactivation of NHE1 has been linked to the development of different pathologies. Several studies in animal models that reproduce the deleterious effects of ischemia/reperfusion injury or cardiac hypertrophy have conclusively demonstrated that NHE1 inhibition provides cardioprotection. Unfortunately, NHE1 inhibitors failed to reproduce these effects in the clinical arena. The reasons for those discrepancies are not apparent yet. However, a reasonable clue to consider would be that drugs that completely abolish the exchanger activity, including that its essential housekeeping function may not be the best therapeutic approach. Therefore, interventions tending to specifically reduce its hyperactive state without affecting its basal activity emerge as a novel potential gold standard. In this regard, a promising goal seems to be the modulation of the phosphorylation state of the cytosolic tail of the exchanger. Recent own experiments demonstrated that Sildenafil, a phosphodiesterase 5A inhibitor drug that has been widely used for the treatment of erectile dysfunction is able to decrease NHE1 phosphorylation, and hence reduce its hyperactivity. In connection, growing evidence demonstrates cardioprotective properties of Sildenafil against different cardiac pathologies, with the distinctive characteristic of directly affecting cardiac tissue without altering blood pressure. This mini-review was aimed to focus on the regulation of NHE1 activity by Sildenafil. For this purpose, experimental data reporting Sildenafil effects in different animal models of heart disease will be discussed.

## Introduction

During the last 40 years, the structure, function, and regulation of the sodium/hydrogen exchanger (NHE) have been deeply studied (1–3). This superfamily of transporter proteins comprises 10 isoforms with different cell localization and physiological roles (4, 5). All isoforms share the common fundamental function of protecting cells from intracellular acidification, by means of an electroneutral elimination of one intracellular H^+^ in exchange for one extracellular Na^+^, and driven by the transmembrane electrochemical Na^+^ gradient (3). This mini review focuses on the ubiquitous isoform 1 (NHE1) of this protein family also known as the “cardiac isoform” and its regulation by the cyclic guanosine monophosphate/protein kinase G (cGMP/PKG) signaling route, triggered by inhibition of the phosphodiesterase 5A (PDE5A) by Sildenafil.

## The Cardiac Na^+^/H^+^ Exchanger “NHE1”

The NHE1 was the first cloned isoform, the most characterized (2, 6, 7), and the main expressed variant found in plasma membrane of mammalian cardiac cells (4). The NHE1 is a dimeric transmembrane protein, and as other NHE isoforms, intracellular H^+^ extrusion through the NHE1 contributes to maintain intracellular pH (pH_i_) of cardiac cells in a healthy narrow range, fulfilling its crucial basal housekeeping function (3). In fact, around normal pH_i_ values of ~7.2, the exchanger activity is low but enough to compensate for basal metabolic H^+^ production. However, under certain conditions, either physiological or pathological, when pH_i_ falls far below the normal range, the NHE1, as well as the sodium-bicarbonate cotransporter, become more active being the NHE1 the dominant alkalizing mechanism (8). Interestingly, the recent demonstration that NHE1 is the isoform expressed in mitochondria (9) suggests a potential role of the exchanger in modulating both mitochondrial Na^+^ and H^+^ gradients (10).

An exacerbated NHE1 activity has been linked to pathological cardiac processes (6). Excessive H^+^ efflux through NHE1 leads to a Na^+^-dependent Ca^2+^ overload with the consequent activation of deleterious routes, including the calcium/calmodulin dependent protein kinase-histone deacetylase signaling pathway (11), and a mitochondrial permeability transition pore (MPTP) opening (9) followed by the release of reactive oxygen species (ROS) and apoptosis (6, 12). Hyperactivity of NHE1 has been found in several diseased states, such as ischemia/reperfusion injury (6) and postischemic cardiac remodeling (6), as well as in pressure overload (13, 14) and hypertensive (15) cardiac hypertrophy. Reinforcing all these evidences, preclinical studies have demonstrated that blunting NHE1 activity effectively provided cardioprotection in different models of heart failure (16–19). In addition, NHE1 blockade during reperfusion of hearts exposed to an ischemic insult promoted preservation of cardiac function as well as minimization of necrosis and/or apoptosis (20, 21). The latter case is rather paradoxical. NHE1 activation after pH_i_ fall during ischemia is necessary to preserve cell integrity. However, extracellular pH lowering due to H^+^ extrusion under reduced blood flow, together with energetic imbalance due to deficient O_2_ supply, rapidly leads to exchanger inhibition. During reperfusion, an immediate washout of extracellular H^+^ suddenly restores NHE1 function whose hyperactivity leads to Na^+^ and Ca^2+^ overload responsible for the deleterious but distinctive effects of this phase (22). The recovery of pH_i_ and the subsequent Ca^+2^ overload results in an increased ROS release due to the MPTP opening, uncontrolled myofibrilar hypercontraction, calpain activation-mediated proteolysis, and finally apoptosis (23).

The promising results of NHE1 inhibition in animal models prompted exploration of the clinical use of NHE1 inhibitors such as cariporide and eniporide in patients. Unfortunately, the results were far from expected, demonstrating lack or insufficient cardioprotection (24, 25) or even worse, severe side effects (26). The reasons for these unexpected discrepancies are not apparent yet. However, the total abolition of the exchanger activity, including both its indispensable function and the pathological hyperactivation, could be a possible explanation. Despite these unforeseen results, inhibition of NHE1 is still considered the most-effective potential therapeutic approach for preventing pathological remodeling of the myocardium irrespective of its origin (27, 28). Thus, a novel scenario of investigation would be to find interventions oriented to specifically reduce the NHE1 hyperactive state. In this regard, a promising approach seems to be the modulation of its phosphorylation level. It is necessary to be reminded that phosphorylation of the cytosolic tail of the NHE1 may either enhance (residues Ser703, Ser770, Ser771) (12, 29) or reduce (Ser 648) (30) exchanger activity. Different kinases have been linked to the phosphorylation/regulation of the NHE1. Among them, the mitogen-activated protein kinase (MAPK) route or the Ras-Raf-MEK-ERK cascade (31) activation in response to different hormones, growth factors, mechanical muscle stretch, or sustained acidosis are the most prominent ones (12, 31–37).

## PDE5A And Cardiac Pathologies: Introducing the PDE5A Inhibitor Sildenafil

Inhibitors of the cGMP-catabolizing enzyme PDE5A are known by their important vasodilatory properties. Sildenafil, among them, was developed as a promising drug against angina pectoris (38) but subsequently safely employed for the treatment of erectile dysfunction (39), pulmonary hypertension, and high-altitude pulmonary edema (40, 41). The main action of Sildenafil was originally assigned to vasodilation and attributed to the increase in the endothelial nitric oxide (NO)-cGMP pathway (42, 43), even in pulmonary hypertension (40). Basal PDE5A expression in cardiac tissue was initially considered insufficient to be detected (44), but further evidence demonstrated that PDE5A is a ubiquitous enzyme (45) and that the myocardium has baseline PDE5A activity (46). Moreover, recent studies showed that upregulation of cardiac PDE5A expression was tightly associated to failing (47, 48) and hypertrophic (49) hearts, to ischemic left and right ventricles (47), and also to the lethal congenital disease known as failing single ventricle (50). In addition, an increased PDE5A expression has been found in different cardiomyopathies that lead to heart failure, such as Chagas (51), or burn-induced (52) cardiac disease. For the specific objective of our current review, PDE5A overexpression has been linked to oxidative stress and the development of pressure overload-triggered myocardial hypertrophy (53, 54), conditions that clearly resemble NHE1 hyperactivation (6). Taken together, the experimental evidence encourages considering that PDE5A inhibition may conceivably be a suitable target to treat cardiac pathologies.

## PDE5A Inhibition By Sildenafil: Effect on Myocardial NHE1

### General Mechanism

The pharmacological target of Sildenafil is PDE5A, with high isozyme selectivity (43). The three PDE5A splicing variants showed equal activity in terms of cGMP-hydrolytic breakdown, as well as similar sensitivity to the inhibition by Sildenafil (45). Due to the structural similarity with cGMP, Sildenafil competitively interacts and inhibits PDE5A enzyme, with the consequent increase in cGMP levels (43). This second messenger binds to PKG, producing an allosteric structural change in the kinase that leads to thiol oxidation, inducing a disulfide homodimer, hence activating the kinase (55). A well-known consequence of PDE5A inhibition is the activation of Ca^+2^-dependent K^+^ channels (BK_Ca_) (43). However, novel actions of Sildenafil will be discussed below.

### Effects of Acute Sildenafil Administration on NHE1 Activity in the Myocardium

**Figure 1:**
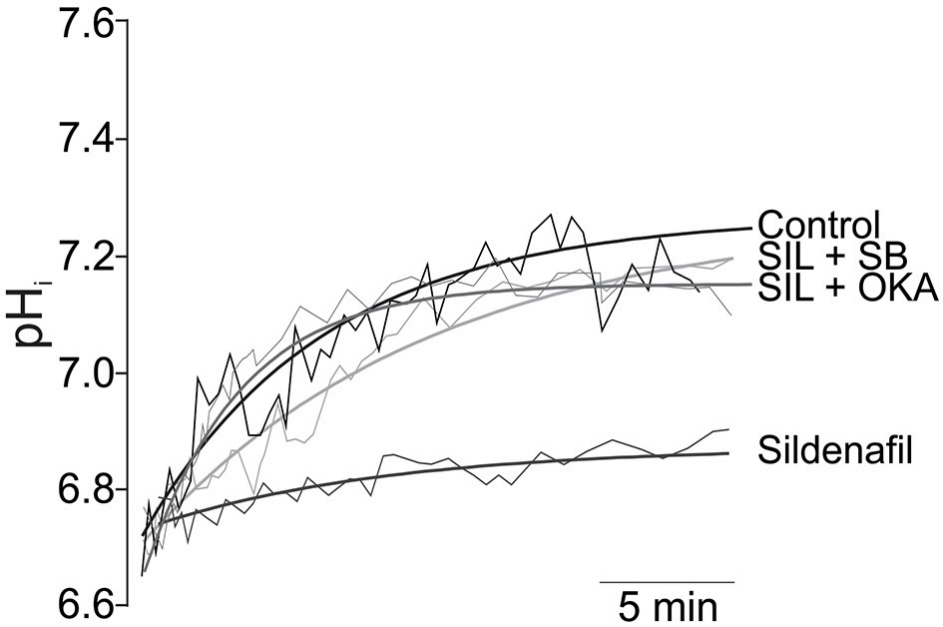
Inhibition of NHE1 activity by Sildenafil. Original traces (thin lines) and its regression approximations (thick lines) of the recovery of pH_i_ after 10 min of sustained acidosis in Control (absence of Sildenafil); 1 mM Sildenafil (SIL); 1 mM Sildenafil plus 10 μM of the p38MAPK inhibitor SB202190 (SIL+SB); and Sildenafil plus 1 nM of Okadaic Acid (SIL+OKA) used to selectively inhibit PP2A. Sildenafil blunted pH_i_ recovery after the acidic challenge, an effect that was reverted either by p38MAPK or PP2A inhibition. Adapted from Díaz et al. (59) with permission from Elsevier.

Sildenafil was found to trigger a direct NHE1 inhibitory action in rat (56, 57) and cat (58) myocardium following sustained intracellular acidosis (Figure 1), and also in the setting of ischemia/reperfusion injury in isolated rat hearts (60). It has been demonstrated that the cGMP/PKG route also regulates different NHE isoforms in different tissues (61, 62). In our hands, inhibition of the NHE1 hyperactivity by acute Sildenafil intervention was due to phosphatase-mediated Ser703 dephosphorylation (57, 58, 60). Importantly, acute NHE1 inhibition by Sildenafil did not affect its basal phosphorylation or expression, and hence preserved its critical homeostatic function (56, 57). This posttranslational regulation of the exchanger was found only after sustained acidosis and was canceled by PKG inhibitors (56). In apparent contradiction to our results, Richards et al. (63) reported two different routes for cGMP-triggered NHE1 regulation according to NO concentration. Using an aminoacidic sequence that reproduced the C-terminal of the NHE1, they showed that high NO concentration triggers PKA-mediated Ser648 phosphorylation, while low NO concentration leads to PKG-mediated Ser703 phosphorylation (63). Although speculative, and thinking in terms of dimeric kinases-mediated phosphorylation (such as PKG and PKA actions), the fact that the authors used part of the whole NHE1 molecule composes a completely different experimental scenario that would condition any possibility of comparison.

**Figure 2:**
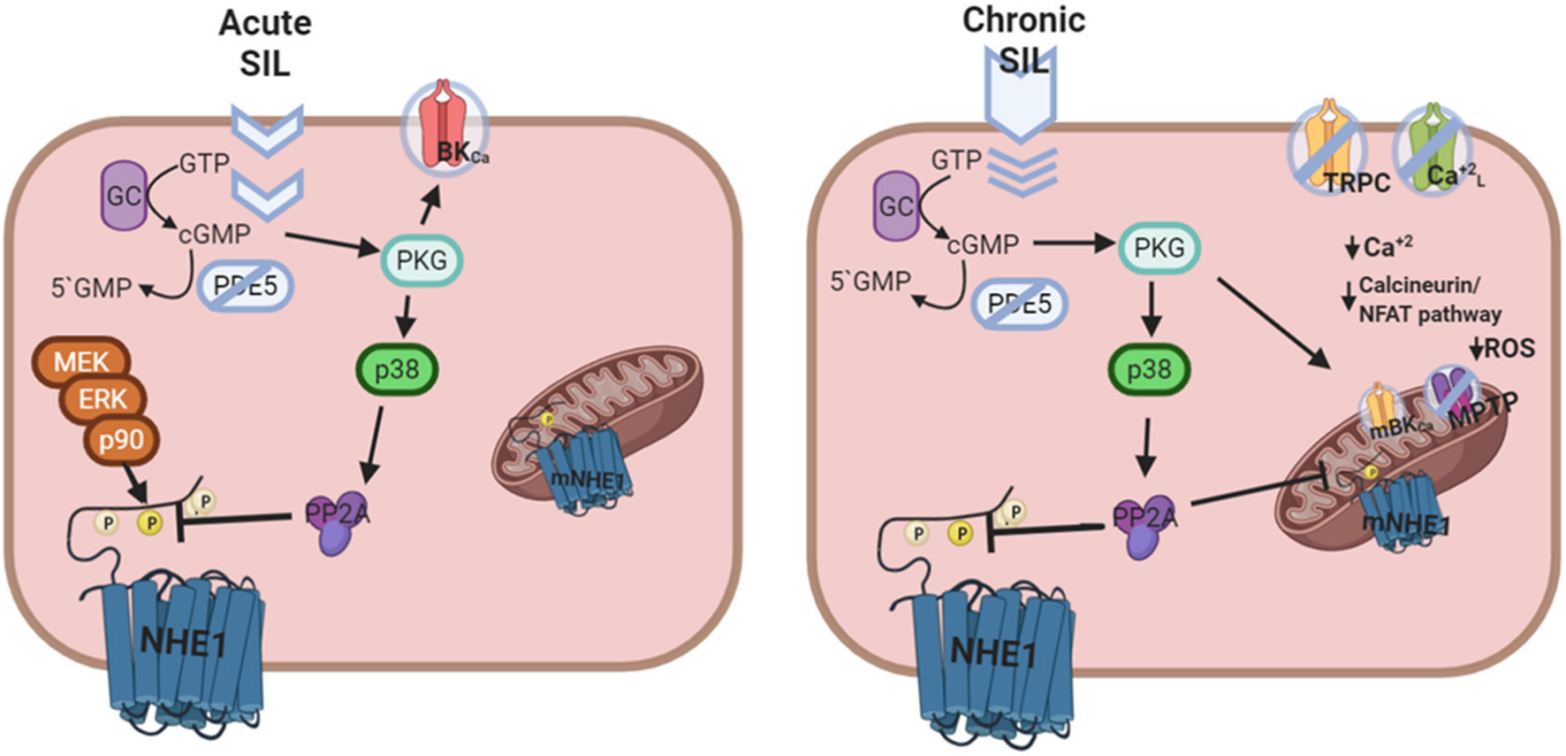
Putative mechanism of cardiac actions of Sildenafil following acute or chronic treatment. Left panel: Acute Sildenafil effects comprise the classical action on BK_Ca_ channels, and the novel cGMP/PKG mediated NHE1 dephosphorylation and inactivation [Adapted from Díaz et al. (59) with permission from Elsevier] involving a sequential activation of p38MAPK and PP2A. Right panel: Chronic Sildenafil effects includes inhibition of TRPC and L-type Ca^+2^ channels, and prevention of NHE1 hyperactivation, all together leading to a decrease in the Ca^+2^ overload-triggered calcineurin/NFAT deleterious pathway. Furthermore, mitochondrial actions of Sildenafil involving mBK_Ca_ channel opening, mNHE1 inhibition, and membrane potential maintenance reduce the probability of MPTP opening and ROS release, contributing to cardioprotection.

We have also demonstrated that the inhibitory effect of Sildenafil on the NHE1 requires activation of the p38MAPK (Figure 1), since blockade of this kinase canceled the inhibitory effect of Sildenafil on the exchanger (59). The exact sequence of events triggered by Sildenafil seems to be as follows: cGMP/PKG activation increase after PDE5A inhibition; p38MAPK phosphorylation/activation by PKG (64); p38MAPK-triggered PP2A assembling (65) and migration to sarcolemmal membrane (66); NHE1 dephosphorylation at Ser703 by PP2A (67); inhibition of NHE1 hyperactivity (57). See Figure 2 for details.

Another controversial subject is the possible role of acute Sildenafil on ROS. In this regard, it has been shown that this drug did not reduce ROS-induced lipid peroxidation in hearts subjected to regional ischemia/reperfusion (60) but reduced mitochondrial H_2_O_2_ production under ischemia (68, 69). Despite possible differences in the sensitivity of experimental methods, ROS-sensitive kinases remained enhanced even after short incubation with Sildenafil, strategy that was enough to effectively inhibit the NHE1 (60). Longer time periods of Sildenafil incubation or preischemic application of the drug conducted to a marked reduction of ROS (68–70).

### Cardiac Intracellular Response to Chronic Sildenafil Administration

Different studies were aimed to explain the subcellular basis of the beneficial actions of chronic PDE5A inhibition by Sildenafil in the myocardium. Among them, it is interesting to recall the described inhibitory effect on voltage-operated L-type Ca^+2^ channels (71), and on the transient receptor potential channels (TRPC) (72), with the consequent decrease in intracellular Ca^2+^, therefore eluding the calcium/calmodulin-NFAT pro-apoptotic pathway (73). The results obtained in our own laboratory add a new piece of knowledge about Sildenafil anti-hypertrophic and anti-apoptotic effect, since its previously unknown inhibitory effect on NHE1 hyperactivity provides an additional clue to understand how this kind of drug can prevent calcium overload. Regarding the modulation of NHE1 activity as a potential therapeutic target, it was reported that complete and chronic blockade of the exchanger triggers protein upregulation to escape from inhibition (74). Interestingly, chronic treatment of infarcted hearts with Sildenafil did not follow this rule by decreasing NHE1 expression (56).

The possibility that chronic effects of Sildenafil could also involve mitochondrial actions should be considered. Chronic Sildenafil treatment improved respiratory rate and reduced ROS production (51). These effects were attributed to restoration of mitochondrial DNA-encoded gene expression (51), activation of mitochondrial BK_Ca_ channels (68), prevention of MPTP opening (75), and/or maintenance of mitochondrial membrane potential (76). In connection, it is important to be reminded that a decrease in mitochondrial NHE1 expression/activity preserves an acidic environment around mitochondria, therefore diminishing Ca^+2^-induced mitochondrial swelling (9). In addition, it was shown that inhibition of MPTP opening leads to a decrease in ROS production after ischemia (77). Considering these results as a whole, the question as to whether Sildenafil could regulate mitochondrial NHE1 certainly arises. In this regard, recent experiments in the hypertrophied myocardium of SHR suggested that a decreased ROS production after chronic Sildenafil treatment may conceivably result from inhibition of mitochondrial NHE1 through a reduction in its phosphorylation state (78). Figure 2 summarizes putative signaling pathways triggered by Sildenafil.

### Cardiac Pharmacodynamics of Sildenafil

As it was mentioned in a previous section, since the approval and commercialization of Sildenafil more than two decades ago (79), the pharmacodynamics of this drug has been extended to different pathological therapeutic targets. Actually, it has been studied for the treatment of different types of cancers (80), Alzheimer disease (81), vascular dementia (82), and also recently proposed to treat COVID-19 patients (83). Regarding cardiac pathologies, Sildenafil showed the ability to prevent and/or reverse cardiac remodeling induced by acute myocardial infarction (56), chronic mitral regurgitation (84), or pressure overload (85, 86). Additional benefits of Sildenafil were attenuation of sympathetic hyperinnervation (87) and promotion of an increase in the QT interval (88), therefore reducing the incidence of ischemia-induced arrhythmia. Concerning to other causes of cardiac morbidity, Sildenafil reverted (89), or prevented (52, 85, 90, 91), cardiac fibrosis development regardless of its origin. Furthermore, improvement of heart failure by PDE5 inhibition was shown to involve attenuation of chronotropic response to dobutamin and a T-tubule network restoration (92). Importantly, different studies under diverse experimental conditions demonstrated that the beneficial cardiac effects of Sildenafil are independent of blood pressure (78, 84, 86).

## Concluding Remarks

We have briefly reviewed the role of Sildenafil on NHE1 regulation and its possible pathophysiological relevance. Classical Sildenafil targets were presented, but the main focus of this revision was centered on the novel inhibitory action of this drug on the NHE1. We were particularly interested in stressing the potential benefits of specifically targeting NHE1 hyperactivity, avoiding its complete blockade, which suggests that Sildenafil treatment would be a better therapeutic approach than traditional NHE1 blockers in the field of cardiology.

Finally, considering that Sildenafil has been safely used during more than two decades (39) and that recent results in heart failure patients are certainly promising (93), we suggest that Sildenafil treatment would represent an appropriate opportunity for immediately access to a less expensive and more secure novel therapeutic alternative to treat severe cardiac pathologies characterized by exacerbated NHE1 activity.

## Author Contributions

All authors listed have made a substantial, direct and intellectual contribution to the work, and approved it for publication.

## Conflict of Interest

The authors declare that the research was conducted in the absence of any commercial or financial relationships that could be construed as a potential conflict of interest.

## References

[1] Aickin CC Thomas RC. An investigation of the ionic mechanism of intracellular pH regulation in mouse soleus muscle fibres. J Physiol. (1977) 273:295–316. 10.1113/jphysiol.1977.sp012095 23428 PMC1353740

[2] Sardet C Franchi A Pouyssegur J. Molecular cloning, primary structure, and expression of the human growth factor-activatable Na+/H+ antiporter. Cell. (1989) 56:271–80. 10.1016/0092-8674(89)90901-X 2536298

[3] Odunewu-Aderibigbe A Fliegel L. The Na(+) /H(+) exchanger and pH regulation in the heart. IUBMB Life. (2014) 66:679–85. 10.1002/iub.1323 25354428

[4] Orlowski J Grinstein S. Diversity of the mammalian sodium/proton exchanger SLC9 gene family. Pflugers Arch. (2004) 447:549–65. 10.1007/s00424-003-1110-3 12845533

[5] Lee SH Kim T Park ES Yang S Jeong D Choi Y. NHE10, an osteoclast-specific member of the Na+/H+ exchanger family, regulates osteoclast differentiation and survival [corrected]. Biochem Biophys Res Commun. (2008) 369:320–6. 10.1016/j.bbrc.2008.01.168 18269914

[6] Karmazyn M Gan XT Humphreys RA Yoshida H Kusumoto K. The myocardial Na(+)-H(+) exchange: structure, regulation, and its role in heart disease. Circ Res. (1999) 85:777–86. 10.1161/01.RES.85.9.777 10532945

[7] Slepkov E Fliegel L. Structure and function of the NHE1 isoform of the Na+/H+ exchanger. Biochem Cell Biol. (2002) 80:499–508. 10.1139/o02-151 12440691

[8] Vaughan-Jones RD Villafuerte FC Swietach P Yamamoto T Rossini A Spitzer KW. pH-Regulated Na(+) influx into the mammalian ventricular myocyte: the relative role of Na(+)-H(+) exchange and Na(+)-HCO Co-transport. J Cardiovasc Electrophysiol. (2006) 17:S134–40. 10.1111/j.1540-8167.2006.00394.x 16686668

[9] Villa-Abrille MC Cingolani E Cingolani HE Alvarez BV. Silencing of cardiac mitochondrial NHE1 prevents mitochondrial permeability transition pore opening. Am J Physiol Heart Circ Physiol. (2011) 300:H1237–51. 10.1152/ajpheart.00840.2010 21297023

[10] Murphy E Eisner DA. Regulation of intracellular and mitochondrial sodium in health and disease. Circ Res. (2009) 104:292–303. 10.1161/CIRCRESAHA.108.189050 19213964 PMC2662399

[11] Nakamura TY Iwata Y Arai Y Komamura K Wakabayashi S. Activation of Na+/H+ exchanger 1 is sufficient to generate Ca2+ signals that induce cardiac hypertrophy and heart failure. Circ Res. (2008) 103:891–9. 10.1161/CIRCRESAHA.108.175141 18776042

[12] Karki P Coccaro E Fliegel L. Sustained intracellular acidosis activates the myocardial Na(+)/H(+) exchanger independent of amino acid Ser(703) and p90(rsk). Biochim Biophys Acta. (2010) 1798:1565–76. 10.1016/j.bbamem.2010.05.005 20471361

[13] Cingolani HE Ennis IL. Sodium-hydrogen exchanger, cardiac overload, and myocardial hypertrophy. Circulation. (2007) 115:1090–100. 10.1161/CIRCULATIONAHA.106.626929 17339567

[14] Mraiche F Oka T Gan XT Karmazyn M Fliegel L. Activated NHE1 is required to induce early cardiac hypertrophy in mice. Basic Res Cardiol. (2011) 106:603–16. 10.1007/s00395-011-0161-4 21359875

[15] Perez NG Alvarez BV Camilion De Hurtado MC Cingolani HE. pHi regulation in myocardium of the spontaneously hypertensive rat. Compensated enhanced activity of the Na(+)-H+ exchanger. Circ Res. (1995) 77:1192–200. 10.1161/01.RES.77.6.1192 7586232

[16] Kusumoto K Haist JV Karmazyn M. Na(+)/H(+) exchange inhibition reduces hypertrophy and heart failure after myocardial infarction in rats. Am J Physiol Heart Circ Physiol. (2001) 280:H738–45. 10.1152/ajpheart.2001.280.2.H738 11158973

[17] Camilion De Hurtado MC Portiansky EL Perez NG Rebolledo OR Cingolani HE. Regression of cardiomyocyte hypertrophy in SHR following chronic inhibition of the Na(+)/H(+) exchanger. Cardiovasc Res. (2002) 53:862–8. 10.1016/S0008-6363(01)00544-2 11922896

[18] Cingolani HE Rebolledo OR Portiansky EL Perez NG Camilion De Hurtado MC. Regression of hypertensive myocardial fibrosis by Na(+)/H(+) exchange inhibition. Hypertension. (2003) 41:373–7. 10.1161/01.HYP.0000051502.93374.1C 12574110

[19] Garciarena CD Caldiz CI Portiansky EL Chiappe De Cingolani GE Ennis IL. Chronic NHE-1 blockade induces an antiapoptotic effect in the hypertrophied heart. J Appl Physiol. (2009) 106:1325–31. 10.1152/japplphysiol.91300.2008 19179646

[20] Humphreys RA Haist JV Chakrabarti S Feng Q Arnold JM Karmazyn M. Orally administered NHE1 inhibitor cariporide reduces acute responses to coronary occlusion and reperfusion. Am J Physiol. (1999) 276:H749–57. 10.1152/ajpheart.1999.276.2.H749 9950878

[21] Gazmuri RJ Radhakrishnan J Ayoub IM. Sodium-hydrogen exchanger isoform-1 inhibition: a promising pharmacological intervention for resuscitation from cardiac arrest. Molecules. (2019) 24:1765. 10.3390/molecules24091765 31067690 PMC6538998

[22] Bobulescu IA Di Sole F Moe OW. Na+/H+ exchangers: physiology and link to hypertension and organ ischemia. Curr Opin Nephrol Hypertens. (2005) 14:485–94. 10.1097/01.mnh.0000174146.52915.5d 16046909 PMC2861558

[23] Garcia-Dorado D Ruiz-Meana M Inserte J Rodriguez-Sinovas A Piper HM. Calcium-mediated cell death during myocardial reperfusion. Cardiovasc Res. (2012) 94:168–80. 10.1093/cvr/cvs116 22499772

[24] Theroux P Chaitman BR Danchin N Erhardt L Meinertz T Schroeder JS. Inhibition of the sodium-hydrogen exchanger with cariporide to prevent myocardial infarction in high-risk ischemic situations. Main results of the GUARDIAN trial Guard during ischemia against necrosis (GUARDIAN) Investigators. Circulation. (2000) 102:3032–8. 10.1161/01.CIR.102.25.3032 11120691

[25] Zeymer U Suryapranata H Monassier JP Opolski G Davies J Rasmanis G. The Na(+)/H(+) exchange inhibitor eniporide as an adjunct to early reperfusion therapy for acute myocardial infarction. Results of the evaluation of the safety and cardioprotective effects of eniporide in acute myocardial infarction (ESCAMI) trial. J Am Coll Cardiol. (2001) 38:1644–50. 10.1016/S0735-1097(01)01608-4 11704395

[26] Mentzer RM Jr Bartels C Bolli R Boyce S Buckberg GD Chaitman B. Sodium-hydrogen exchange inhibition by cariporide to reduce the risk of ischemic cardiac events in patients undergoing coronary artery bypass grafting: results of the EXPEDITION study. Ann Thorac Surg. (2008) 85:1261–70. 10.1016/j.athoracsur.2007.10.054 18355507

[27] Karmazyn M. NHE-1: still a viable therapeutic target. J Mol Cell Cardiol. (2013) 61:77–82. 10.1016/j.yjmcc.2013.02.006 23429008

[28] Medina AJ Pinilla OA Portiansky EL Caldiz CI Ennis IL. Silencing of the Na(+)/H(+) exchanger 1(NHE-1) prevents cardiac structural and functional remodeling induced by angiotensin II. Exp Mol Pathol. (2019) 107:1–9. 10.1016/j.yexmp.2019.01.007 30664842

[29] Avkiran M Haworth RS. Regulatory effects of G protein-coupled receptors on cardiac sarcolemmal Na+/H+ exchanger activity: signalling and significance. Cardiovasc Res. (2003) 57:942–52. 10.1016/S0008-6363(02)00782-4 12650872

[30] Snabaitis AK Cuello F Avkiran M. Protein kinase B/Akt phosphorylates and inhibits the cardiac Na+/H+ exchanger NHE1. Circ Res. (2008) 103:881–90. 10.1161/CIRCRESAHA.108.175877 18757828

[31] Gallo S Vitacolonna A Bonzano A Comoglio P Crepaldi T. ERK: a key player in the pathophysiology of cardiac hypertrophy. Int J Mol Sci. (2019) 20:2164. 10.3390/ijms20092164 31052420 PMC6539093

[32] Berk BC Aronow MS Brock TA Cragoe E Jr Gimbrone MA Jr Alexander RW. Angiotensin II-stimulated Na+/H+ exchange in cultured vascular smooth muscle cells. Evidence for protein kinase C-dependent and -independent pathways. J Biol Chem. (1987) 262:5057–64. 10.1016/S0021-9258(18)61153-6 3031037

[33] Ebata S Muto S Okada K Nemoto J Amemiya M Saito T. Aldosterone activates Na+/H+ exchange in vascular smooth muscle cells by nongenomic and genomic mechanisms. Kidney Int. (1999) 56:1400–12. 10.1046/j.1523-1755.1999.00674.x 10504492

[34] Takahashi E Abe J Gallis B Aebersold R Spring DJ Krebs EG. p90(RSK) is a serum-stimulated Na+/H+ exchanger isoform-1 kinase. Regulatory phosphorylation of serine 703 of Na+/H+ exchanger isoform-1. J Biol Chem. (1999) 274:20206–14. 10.1074/jbc.274.29.20206 10400637

[35] Haworth RS Mccann C Snabaitis AK Roberts NA Avkiran M. Stimulation of the plasma membrane Na+/H+ exchanger NHE1 by sustained intracellular acidosis. Evidence for a novel mechanism mediated by the ERK pathway. J Biol Chem. (2003) 278:31676–84. 10.1074/jbc.M304400200 12791686

[36] Perez NG Villa-Abrille MC Aiello EA Dulce RA Cingolani HE Camilion De Hurtado MC. A low dose of angiotensin II increases inotropism through activation of reverse Na(+)/Ca(2+) exchange by endothelin release. Cardiovasc Res. (2003) 60:589–97. 10.1016/j.cardiores.2003.09.004 14659804

[37] Coaxum SD Garnovskaya MN Gooz M Baldys A Raymond JR. Epidermal growth factor activates Na(+/)H(+) exchanger in podocytes through a mechanism that involves Janus kinase and calmodulin. Biochim Biophys Acta. (2009) 1793:1174–81. 10.1016/j.bbamcr.2009.03.006 19341767 PMC3364543

[38] Jackson G Montorsi P Cheitlin MD. Cardiovascular safety of sildenafil citrate (Viagra): an updated perspective. Urology. (2006) 68:47–60. 10.1016/j.urology.2006.05.047 17011375

[39] Conti CR Pepine CJ Sweeney M. Efficacy and safety of sildenafil citrate in the treatment of erectile dysfunction in patients with ischemic heart disease. Am J Cardiol. (1999) 83:29C−34C. 10.1016/S0002-9149(99)00045-4 10078540

[40] Ladha F Bonnet S Eaton F Hashimoto K Korbutt G Thebaud B. Sildenafil improves alveolar growth and pulmonary hypertension in hyperoxia-induced lung injury. Am J Respir Crit Care Med. (2005) 172:750–6. 10.1164/rccm.200503-510OC 15947285

[41] Bates MG Thompson AA Baillie JK Sutherland AI Irving JB Hirani N. Sildenafil citrate for the prevention of high altitude hypoxic pulmonary hypertension: double blind, randomized, placebo-controlled trial. High Alt Med Biol. (2011) 12:207–14. 10.1089/ham.2011.0007 21962063

[42] Glossmann H Petrischor G Bartsch G. Molecular mechanisms of the effects of sildenafil (VIAGRA). Exp Gerontol. (1999) 34:305–18. 10.1016/S0531-5565(99)00003-0 10433386

[43] Corbin JD Francis SH. Molecular biology and pharmacology of PDE-5-inhibitor therapy for erectile dysfunction. J Androl. (2003) 24:S38–41. 10.1002/j.1939-4640.2003.tb02744.x 14581493

[44] Degen CV Bishu K Zakeri R Ogut O Redfield MM Brozovich FV. The emperor's new clothes: PDE5 and the heart. PLoS ONE. (2015) 10:e0118664. 10.1371/journal.pone.0118664 25747598 PMC4351884

[45] Lin CS. Tissue expression, distribution, and regulation of PDE5. Int J Impot Res. (2004) 16:S8–10. 10.1038/sj.ijir.3901207 15224128

[46] Zhang M Koitabashi N Nagayama T Rambaran R Feng N Takimoto E. Expression, activity, and pro-hypertrophic effects of PDE5A in cardiac myocytes. Cell Signal. (2008) 20:2231–6. 10.1016/j.cellsig.2008.08.012 18790048 PMC2601628

[47] Pokreisz P Vandenwijngaert S Bito V Van Den Bergh A Lenaerts I Busch C. Ventricular phosphodiesterase-5 expression is increased in patients with advanced heart failure and contributes to adverse ventricular remodeling after myocardial infarction in mice. Circulation. (2009) 119:408–16. 10.1161/CIRCULATIONAHA.108.822072 19139381 PMC3791110

[48] Shan X Quaile MP Monk JK French B Cappola TP Margulies KB. Differential expression of PDE5 in failing and nonfailing human myocardium. Circ Heart Fail. (2012) 5:79–86. 10.1161/CIRCHEARTFAILURE.111.961706 22135403 PMC3261338

[49] Vandenwijngaert S Pokreisz P Hermans H Gillijns H Pellens M Bax NA. Increased cardiac myocyte PDE5 levels in human and murine pressure overload hypertrophy contribute to adverse LV remodeling. PLoS ONE. (2013) 8:e58841. 10.1371/journal.pone.0058841 23527037 PMC3601083

[50] Garcia AM Nakano SJ Karimpour-Fard A Nunley K Blain-Nelson P Stafford NM. Phosphodiesterase-5 is elevated in failing single ventricle myocardium and affects cardiomyocyte remodeling *in vitro*. Circ Heart Fail. (2018) 11:e004571. 10.1161/CIRCHEARTFAILURE.117.004571 30354365 PMC6206883

[51] Wen JJ Wan X Thacker J Garg NJ. Chemotherapeutic efficacy of phosphodiesterase inhibitors in chagasic cardiomyopathy. JACC Basic Transl Sci. (2016) 1:235–50. 10.1016/j.jacbts.2016.04.005 27747306 PMC5065248

[52] Wen JJ Cummins C Radhakrishnan RS. Sildenafil recovers burn-induced cardiomyopathy. Cells. (2020) 9:1393. 10.3390/cells9061393 PMC7349507 32503314

[53] Lu Z Xu X Hu X Lee S Traverse JH Zhu G. Oxidative stress regulates left ventricular PDE5 expression in the failing heart. Circulation. (2010) 121:1474–83. 10.1161/CIRCULATIONAHA.109.906818 20308615 PMC3110701

[54] Zhang M Takimoto E Hsu S Lee DI Nagayama T Danner T. Myocardial remodeling is controlled by myocyte-targeted gene regulation of phosphodiesterase type 5. J Am Coll Cardiol. (2010) 56:2021–30. 10.1016/j.jacc.2010.08.612 20970280 PMC3036840

[55] Wall ME Francis SH Corbin JD Grimes K Richie-Jannetta R Kotera J. Mechanisms associated with cGMP binding and activation of cGMP-dependent protein kinase. Proc Natl Acad Sci USA. (2003) 100:2380–5. 10.1073/pnas.0534892100 12591946 PMC151349

[56] Perez NG Piaggio MR Ennis IL Garciarena CD Morales C Escudero EM. Phosphodiesterase 5A inhibition induces Na+/H+ exchanger blockade and protection against myocardial infarction. Hypertension. (2007) 49:1095–103. 10.1161/HYPERTENSIONAHA.107.087759 17339532

[57] Diaz RG Nolly MB Massarutti C Casarini MJ Garciarena CD Ennis IL. Phosphodiesterase 5A inhibition decreases NHE-1 activity without altering steady state pH(i): role of phosphatases. Cell Physiol Biochem. (2010) 26:531–40. 10.1159/000322321 21063091

[58] Yeves AM Garciarena CD Nolly MB Chiappe De Cingolani GE Cingolani HE Ennis IL. Decreased activity of the Na+/H+ exchanger by phosphodiesterase 5A inhibition is attributed to an increase in protein phosphatase activity. Hypertension. (2010) 56:690–5. 10.1161/HYPERTENSIONAHA.110.151324 20713918

[59] Diaz RG Escudero DS Brea MS Morgan PE Perez NG. p38 mitogen activated protein kinase mediates cardiac Na+/H+ exchanger inhibition induced by Sildenafil. Eur J Pharmacol. (2019) 849:96–105. 10.1016/j.ejphar.2019.01.070 30721701

[60] Garciarena CD Fantinelli JC Caldiz CI Chiappe De Cingolani G Ennis IL Perez NG. Myocardial reperfusion injury: reactive oxygen species vs. NHE-1 reactivation. Cell Physiol Biochem. (2011) 27:13–22. 10.1159/000325201 21325817

[61] Schulte EA Hohendahl A Stegemann H Hirsch JR Saleh H Schlatter E. Natriuretic peptides and diadenosine polyphosphates modulate pH regulation of rat mesangial cells. Cell Physiol Biochem. (1999) 9:310–22. 10.1159/000016325 10749997

[62] Gill RK Saksena S Syed IA Tyagi S Alrefai WA Malakooti J. Regulation of NHE3 by nitric oxide in Caco-2 cells. Am J Physiol Gastrointest Liver Physiol. (2002) 283:G747–56. 10.1152/ajpgi.00294.2001 12181191

[63] Richards MA Simon JN Ma R Loonat AA Crabtree MJ Paterson DJ. Nitric oxide modulates cardiomyocyte pH control through a biphasic effect on sodium/hydrogen exchanger-1. Cardiovasc Res. (2020) 116:1958–71. 10.1093/cvr/cvz311 31742355 PMC7567331

[64] Li Z Zhang G Feil R Han J Du X. Sequential activation of p38 and ERK pathways by cGMP-dependent protein kinase leading to activation of the platelet integrin alphaIIb beta3. Blood. (2006) 107:965–72. 10.1182/blood-2005-03-1308 16210341 PMC1464421

[65] Liu Q Hofmann PA. Modulation of protein phosphatase 2a by adenosine A1 receptors in cardiomyocytes: role for p38 MAPK. Am J Physiol Heart Circ Physiol. (2003) 285:H97–103. 10.1152/ajpheart.00956.2002 12649078

[66] Zuluaga S Alvarez-Barrientos A Gutierrez-Uzquiza A Benito M Nebreda AR Porras A. Negative regulation of Akt activity by p38alpha MAP kinase in cardiomyocytes involves membrane localization of PP2A through interaction with caveolin-1. Cell Signal. (2007) 19:62–74. 10.1016/j.cellsig.2006.05.032 16844343

[67] Snabaitis AK D'mello R Dashnyam S Avkiran M. A novel role for protein phosphatase 2A in receptor-mediated regulation of the cardiac sarcolemmal Na+/H+ exchanger NHE1. J Biol Chem. (2006) 281:20252–62. 10.1074/jbc.M600268200 16707501

[68] Ockaili R Salloum F Hawkins J Kukreja RC. Sildenafil (Viagra) induces powerful cardioprotective effect via opening of mitochondrial K(ATP) channels in rabbits. Am J Physiol Heart Circ Physiol. (2002) 283:H1263–9. 10.1152/ajpheart.00324.2002 12181158

[69] Zaobornyj T Mazo T Perez V Gomez A Contin M Tripodi V. Thioredoxin-1 is required for the cardioprotecive effect of sildenafil against ischaemia/reperfusion injury and mitochondrial dysfunction in mice. Free Radic Res. (2019) 53:993–1004. 10.1080/10715762.2019.1661404 31455116

[70] Behmenburg F Dorsch M Huhn R Mally D Heinen A Hollmann MW. Impact of mitochondrial Ca2+-sensitive potassium (mBKCa) channels in sildenafil-induced cardioprotection in rats. PLoS ONE. (2015) 10:e0144737. 10.1371/journal.pone.0144737 26671662 PMC4684397

[71] Chiang CE Luk HN Wang TM Ding PY. Effects of sildenafil on cardiac repolarization. Cardiovasc Res. (2002) 55:290–9. 10.1016/S0008-6363(02)00438-8 12123768

[72] Koitabashi N Aiba T Hesketh GG Rowell J Zhang M Takimoto E. Cyclic GMP/PKG-dependent inhibition of TRPC6 channel activity and expression negatively regulates cardiomyocyte NFAT activation Novel mechanism of cardiac stress modulation by PDE5 inhibition. J Mol Cell Cardiol. (2010) 48:713–24. 10.1016/j.yjmcc.2009.11.015 19961855 PMC2837762

[73] Fiedler B Lohmann SM Smolenski A Linnemuller S Pieske B Schroder F. Inhibition of calcineurin-NFAT hypertrophy signaling by cGMP-dependent protein kinase type I in cardiac myocytes. Proc Natl Acad Sci USA. (2002) 99:11363–8. 10.1073/pnas.162100799 12177418 PMC123262

[74] Camilion De Hurtado MC Ennis IL Perez NG Chiappe De Cingolani GE Morgan P. Upregulation of myocardial Na+/H+ exchanger induced by chronic treatment with a selective inhibitor. J Mol Cell Cardiol. (2002) 34:1539–47. 10.1006/jmcc.2002.2107 12431452

[75] Ascah A Khairallah M Daussin F Bourcier-Lucas C Godin R Allen BG. Stress-induced opening of the permeability transition pore in the dystrophin-deficient heart is attenuated by acute treatment with sildenafil. Am J Physiol Heart Circ Physiol. (2011) 300:H144–53. 10.1152/ajpheart.00522.2010 20971771

[76] Fisher PW Salloum F Das A Hyder H Kukreja RC. Phosphodiesterase-5 inhibition with sildenafil attenuates cardiomyocyte apoptosis and left ventricular dysfunction in a chronic model of doxorubicin cardiotoxicity. Circulation. (2005) 111:1601–10. 10.1161/01.CIR.0000160359.49478.C2 15811867

[77] Javadov S Karmazyn M. Mitochondrial permeability transition pore opening as an endpoint to initiate cell death and as a putative target for cardioprotection. Cell Physiol Biochem. (2007) 20:1–22. 10.1159/000103747 17595511

[78] Escudero DS Brea MS Caldiz CI Amarillo ME Aranda JO Portiansky EL. PDE5 inhibition improves cardiac morphology and function in SHR by reducing NHE1 activity: repurposing Sildenafil for the treatment of hypertensive cardiac hypertrophy. Eur J Pharmacol. (2020) 891:173724. 10.1016/j.ejphar.2020.173724 33152335

[79] Delate T Simmons VA Motheral BR. Patterns of use of sildenafil among commercially insured adults in the United States: 1998-2002. Int J Impot Res. (2004) 16:313–8. 10.1038/sj.ijir.3901191 14973524

[80] Pantziarka P Sukhatme V Crispino S Bouche G Meheus L Sukhatme VP. Repurposing drugs in oncology (ReDO)-selective PDE5 inhibitors as anti-cancer agents. Ecancermedicalscience. (2018) 12:824. 10.3332/ecancer.2018.824 29743944 PMC5931815

[81] Zuccarello E Acquarone E Calcagno E Argyrousi EK Deng SX Landry DW. Development of novel phosphodiesterase 5 inhibitors for the therapy of Alzheimer's disease. Biochem Pharmacol. (2020) 176:113818. 10.1016/j.bcp.2020.113818 31978378 PMC7263960

[82] Venkat P Chopp M Zacharek A Cui C Landschoot-Ward J Qian Y. Sildenafil treatment of vascular dementia in aged rats. Neurochem Int. (2019) 127:103–12. 10.1016/j.neuint.2018.12.015 30592970

[83] Mondaini N. Phosphodiesterase type 5 inhibitors and COVID-19: are they useful in disease management? World J Mens Health. (2020) 38:254–5. 10.5534/wjmh.200089 32573126 PMC7308240

[84] Kim KH Kim YJ Ohn JH Yang J Lee SE Lee SW. Long-term effects of sildenafil in a rat model of chronic mitral regurgitation: benefits of ventricular remodeling and exercise capacity. Circulation. (2012) 125:1390–401. 10.1161/CIRCULATIONAHA.111.065300 22319106

[85] Takimoto E Champion HC Li M Belardi D Ren S Rodriguez ER. Chronic inhibition of cyclic GMP phosphodiesterase 5A prevents and reverses cardiac hypertrophy. Nat Med. (2005) 11:214–22. 10.1038/nm1175 15665834

[86] Westermann D Becher PM Lindner D Savvatis K Xia Y Frohlich M. Selective PDE5A inhibition with sildenafil rescues left ventricular dysfunction, inflammatory immune response and cardiac remodeling in angiotensin II-induced heart failure *in vivo*. Basic Res Cardiol. (2012) 107:308. 10.1007/s00395-012-0308-y 23117837

[87] Lee TM Chen CC Chung TH Chang NC. Effect of sildenafil on ventricular arrhythmias in post-infarcted rat hearts. Eur J Pharmacol. (2012) 690:124–32. 10.1016/j.ejphar.2012.05.015 22683410

[88] Nagy O Hajnal A Parratt JR Vegh A. Sildenafil (Viagra) reduces arrhythmia severity during ischaemia 24 h after oral administration in dogs. Br J Pharmacol. (2004) 141:549–51. 10.1038/sj.bjp.0705658 14744808 PMC1574240

[89] Nakamura T Zhu G Ranek MJ Kokkonen-Simon K Zhang M Kim GE. Prevention of PKG-1alpha oxidation suppresses antihypertrophic/antifibrotic effects from PDE5 inhibition but not sGC stimulation. Circ Heart Fail. (2018) 11:e004740. 10.1161/CIRCHEARTFAILURE.117.004740 29545395 PMC5858464

[90] Gong W Yan M Chen J Chaugai S Chen C Wang D. Chronic inhibition of cyclic guanosine monophosphate-specific phosphodiesterase 5 prevented cardiac fibrosis through inhibition of transforming growth factor beta-induced Smad signaling. Front Med. (2014) 8:445–55. 10.1007/s11684-014-0378-3 25416030

[91] Patrucco E Domes K Sbroggio M Blaich A Schlossmann J Desch M. Roles of cGMP-dependent protein kinase I (cGKI) and PDE5 in the regulation of Ang II-induced cardiac hypertrophy and fibrosis. Proc Natl Acad Sci USA. (2014) 111:12925–9. 10.1073/pnas.1414364111 25139994 PMC4156763

[92] Lawless M Caldwell JL Radcliffe EJ Smith CER Madders GWP Hutchings DC. Phosphodiesterase 5 inhibition improves contractile function and restores transverse tubule loss and catecholamine responsiveness in heart failure. Sci Rep. (2019) 9:6801. 10.1038/s41598-019-42592-1 31043634 PMC6494852

[93] Guazzi M Vicenzi M Arena R Guazzi MD. PDE5 inhibition with sildenafil improves left ventricular diastolic function, cardiac geometry, and clinical status in patients with stable systolic heart failure: results of a 1-year, prospective, randomized, placebo-controlled study. Circ Heart Fail. (2011) 4:8–17. 10.1161/CIRCHEARTFAILURE.110.944694 21036891

